# Work meaning in medicine: a comparative investigation across training stages

**DOI:** 10.3389/fmed.2026.1780736

**Published:** 2026-04-29

**Authors:** Nicole Kovaks, Yuliya Lipshits-Braziler, Sima Amram-Vaknin, Tony Gutentag

**Affiliations:** 1 Gray Faculty of Medical and Health Sciences, Tel Aviv University, Tel Aviv-Yafo, Israel; 2 The Seymour Fox School of Education, The Hebrew University of Jerusalem, Jerusalem, Israel

**Keywords:** burnout, calling, medical education, medicine, work meaning, work orientations, work-related wellbeing

## Abstract

**Introduction:**

Work orientations (i.e., the meaning individuals attribute to the purpose of their work) are established predictors of work-related wellbeing in the general population. This investigation examined an expanded model of work orientations (i.e., calling, job, career, social embeddedness, and busyness) in the medical profession. It compared physicians, residents, and clinical-year medical students across six indicators for work-related wellbeing: person-environment fit, work satisfaction, work engagement, burnout, turnover intentions, and commitment to the profession.

**Materials and methods:**

A cross-sectional survey was administered to 198 participants from Israel, including physicians, residents, and clinical-year medical students. Measures included a work orientations questionnaire, indicators of work-related wellbeing, and a demographic questionnaire. Group differences were examined using analyses of variance (ANOVAs), and Pearson correlations were computed to assess associations between work orientations and work-related wellbeing indicators.

**Results:**

Calling was the most strongly endorsed orientation across all groups. Medical students reported higher calling than residents, and higher career and social embeddedness than physicians. Calling was most consistently associated with work-related wellbeing across the stages of the medical profession, whereas the associations of social embeddedness and busyness with wellbeing were most notable among residents and students.

**Discussion:**

These findings underscore the developmental nature of the associations between work orientations and work-related wellbeing in medicine, offering implications for both theory and practice.

## 1 Introduction

Work constitutes a central aspect of modern life, and in today's unpredictable world, finding meaning and purpose in work has become increasingly important (1). The way individuals perceive the purpose of their work can be described through an expanded model of five orientations: calling, job, career, social embeddedness, and busyness (2, 3). A growing body of research has shown that these orientations are associated with key indicators of work-related wellbeing, including work satisfaction and work engagement (2–5). The medical profession, with its intense demands and high responsibility, places work at the center of practitioners' lives (6, 7), making work orientation especially relevant in this context. However, the expanded model of work orientations has not yet been examined in the medical field, and existing studies have typically focused on a limited range of work-related wellbeing indicators. The present investigation addresses this gap by employing the expanded five-dimensional model of work orientations and a wide range of work-related wellbeing indicators, specifically in relation to the medical profession.

Work orientation refers to individuals' beliefs about the purpose of work in their lives and its role in providing meaning (2, 8, 9). The tripartite model of work orientations, initially conceptualized by Bellah et al. (8) and later operationalized by Wrzesniewski et al. (9), has served as a dominant framework in the field for several decades. This model delineates three primary work orientations: calling, job, and career (9). Those with a calling orientation perceive their work as their life's mission and purpose, a source of enjoyment, and a means to make the world a better place. Those with a job orientation perceive their work as a means to gain financial stability and support themselves and their families, enabling them to lead their lives outside of work. Finally, those with a career orientation perceive their work as a means of advancing within the hierarchy in their field and gaining influence, responsibility and responsibility over their coworkers (9).

Building on this foundational framework, Willner et al. (2) proposed an expanded model, introducing two additional dimensions: social embeddedness and busyness, resulting in a more comprehensive five-dimensional conceptualization of work orientations. Those with a social embeddedness orientation perceive their work as a means to connect with people, establish relationships beyond the workplace, and be a part of a team or a family-like group. Those with a busyness orientation perceive their work as a means to spend their days. Without their work, they feel like their day moves slowly, filled with boredom and emptiness (2). This expanded model has received empirical support in samples of employed adults across diverse professions (2–5). However, it has not yet been tested within the medical field.

Previous research has examined associations between work orientations and selected indicators of work-related wellbeing, such as work satisfaction and work engagement (2, 4, 5). The present investigation extends previous research by exploring a broader range of work-related wellbeing indicators: person-environment fit, work satisfaction, work engagement, burnout, turnover intentions, and commitment to the profession.

Person-environment fit refers to the compatibility between a person's characteristics or preferences (e.g., values, personality, goals) and their working environment (10). Higher person-environment fit is associated with greater satisfaction and commitment to the profession, as well as lower turnover intentions (10). Work satisfaction is a pleasurable emotional state resulting from evaluating one's job as fulfilling one's job values (11). Higher work satisfaction is associated with greater work engagement, resilience, reduced turnover intentions, and work performance (12, 13). Work engagement is a positive, fulfilling work-related state of mind characterized by vigor, dedication, and absorption in work (14). Greater work engagement indicates work-related wellbeing as engaged workers are happier, perform better, and are more satisfied with both their work and their lives (15). Burnout is a work-related state of exhaustion that occurs among employees, characterized by extreme tiredness, reduced ability to regulate cognitive and emotional processes, and mental distancing (16). Burnout has been linked to lower work engagement, job satisfaction and commitment, as well as higher turnover intentions, psychological distress, and depression (17). Turnover refers to the rate at which employees leave a position and are subsequently replaced. Moreover, physicians' turnover intentions have been found to predict actual turnover (18). Finally, commitment to the profession is the degree of psychological attachment to the profession (19). Greater commitment has been associated with lower burnout and higher work engagement (20).

In the present investigation, we examined whether and to what extent work orientations are associated with multiple indicators of work-related wellbeing across various stages of professional development in medicine: physicians, residents, and clinical-year medical students. We focused on medicine because, in this profession, work constitutes a substantial part of workers' lives, both in terms of time investment and its central role in professional identity (6, 7). Consequently, work orientations are likely to be especially pronounced, making this profession a compelling context for study. Moreover, in medicine, workers' wellbeing is often compromised. Physicians face high rates of burnout, strong intentions to leave their current practice, reduced life satisfaction, and increased stress and depression [e.g., (21–24)]. Given the essential role physicians play in the healthcare system, it is critical to identify factors associated with their work-related wellbeing.

Although work orientations have been explored more extensively in other professions, research in medicine remains limited. Studies in other fields have shown that certain work orientations predicted work satisfaction (2, 5), work engagement (4, 5), and turnover intentions (25). To offer a more holistic perspective, our investigation moves beyond these indicators to include additional work-related wellbeing indicators: person-environment fit, burnout, and commitment to the profession. Additionally, it examines their associations with all five work orientations across the various stages of the medical profession.

A further contribution of this investigation is its focus on different stages of medical training and practice. Previous research has typically examined only one group, either physicians, residents, or medical students [e.g., (26–28)]. Consequently, relatively few studies have compared some or all three of these populations [e.g., (29, 30)]. The present investigation addresses this gap, by examining and comparing these groups with respect to their work orientations, work-related wellbeing, and the associations between these constructs.

Accumulating evidence suggests that work orientations are systematically associated with work-related wellbeing. Among the five orientations, a calling orientation has demonstrated the most consistent positive associations with work-related wellbeing. In the general population and among healthcare professionals, including nurses, calling has been associated with higher person-environment fit (31), greater work satisfaction (2, 4, 32), stronger work engagement (4, 5), lower burnout (33, 34), and fewer turnover intentions (25, 32, 35). Similar patterns have been observed in specific medical populations, where calling has been associated with higher work satisfaction among physicians (36) and medical students (37) and lower burnout among physicians (7, 38). We therefore hypothesized that calling-oriented individuals would report higher person-environment fit, work satisfaction, and work engagement, alongside lower burnout and turnover intentions.

In contrast, job orientation has generally been associated with less favorable outcomes. In the general population, it has been associated with lower work satisfaction (2, 4, 5) and lower work engagement (4, 5), though it has not been consistently related to turnover intentions (25). We therefore hypothesized that job-oriented individuals would report lower work satisfaction and work engagement.

While previous research in the general population has not found significant associations between career orientation and work satisfaction (2, 4) or work engagement (4), career orientation has been associated with higher turnover intentions (25). We therefore hypothesized that career-oriented individuals would report higher turnover intentions.

Beyond the traditional tripartite model, research on social embeddedness indicates that this orientation was associated with higher work satisfaction (2, 4) and greater work engagement (4, 5) in the general population, and lower burnout among nurses (39). Thus, we hypothesized that social embeddedness-oriented individuals would report higher work satisfaction and work engagement and lower burnout.

Finally, busyness orientation has been associated with lower work satisfaction in the general population (2). Findings regarding work engagement were inconsistent, with some research reporting no association (4) and others identifying a positive association (5). We therefore hypothesized that busyness-oriented individuals would report lower work satisfaction.

Although previous research has demonstrated that work orientations are associated with work-related wellbeing, most studies have relied on the traditional three-dimensional model (calling, job, career), examined a limited set of work-related wellbeing outcomes, and focused primarily on non-medical populations. The present investigation applies the expanded five-dimensional model of work orientations and a broad set of work-related wellbeing indicators within the medical profession. Specifically, we examine and compare physicians, residents, and medical students in their clinical years.

## 2 Materials and methods

### 2.1 Participants

The sample comprised 198 participants from Israel, including physicians (*n*_1_ = 54; *M*_*age*_ = 43.46, *SD*_*age*_ = 9.56; 78% females; *M*_seniority_ = 13.75, *SD*_seniority_ = 10.79), residents (*n*_2_ = 62; *M*_*age*_ = 35.48, *SD*_*age*_ = 4.90; 53% females; *M*_*residency*_*year*_ = 2.66, *SD*_*residency*_*year*_ = 1.53), and clinical-year medical students (*n*_3_ = 82; *M*_*age*_ = 28.35, *SD*_*age*_ = 3.23; 62% females; *M*_*school*_*year*_ = 4.61, *SD*_*school*_*year*_ = 1.17). Inclusion criteria were being a physician, resident, or medical student in their clinical years. Individuals who did not meet these criteria were automatically screened out of the online questionnaire.

Participants were recruited via social media platforms (e.g., Facebook) and WhatsApp, targeting medical professionals and students. As an incentive, participants were offered the opportunity to enter a lottery in which four participants were randomly selected to receive a US$55.96 prize. A power analysis conducted using G^*^Power 3.0 (40) indicated that a sample of 193 was required to detect a small effect (*r* = 0.20; 1–β = 0.80; α = 0.05). The final sample exceeded this requirement.

### 2.2 Instruments

#### 2.2.1 Work orientation questionnaire (WOQ)

The WOQ (2) assesses the meaning individuals give to their work or the meaning that their work has in their life in terms of five dimensions: calling, job, career, social embeddedness, and busyness. The original version comprises 26 items with five statements representing each of the five dimensions and a warm-up item (“What I do for work is very important to me”). We used the short form of the WOQ (41), an unpublished measure developed by one of the authors of the present investigation, to provide a more economical assessment of the five work orientations. The short version comprises 18 items: three statements for each of the five dimensions, one warm-up item, and two validity items (“I expect to receive fair treatment at work”; “I don't care where or what I work in”). Sample items for five work orientations include: “My work makes the world a better place” (calling); “My main reason for working is to earn a living that will allow me to lead my life outside of work” (job); “I hope to gain influence and power in my workplace” (career); “The people I work with are like a family to me” (social embeddedness); and “I dislike having nothing to do, so I prefer to work” (busyness). Participants were asked to rate on a seven-point Likert-type scale the degree to which each item describes them, ranging from 1 (*not at all*) to 7 (*very much*). Based on a sample of 230 Israeli employed adults, Willner et al. (41) reported a median Cronbach alpha (Cα) internal-consistency reliability of 0.82 for the five scales of the short form (range 0.73–0.88). In addition, the short form demonstrated a similar overall pattern of associations with work satisfaction, providing preliminary evidence of criterion-related validity. The Cα internal-consistency reliability estimates of the five orientation scales in the present investigation were as follows: for calling, 0.77 for physicians, 0.51 for residents, 0.68 for students, and 0.67 overall; for job, 0.92, 0.88, 0.87, and 0.89, respectively; for career, 0.88, 0.68, 0.83, and 0.82, respectively; for social embeddedness, 0.57, 0.70, 0.70, and 0.68, respectively; and for busyness, 0.76, 0.64, 0.76, and 0.74, respectively.

#### 2.2.2 Person-environment fit

Person–environment fit was assessed using a single-item measure (41). Participants were asked to indicate the extent to which there was a fit between the meaning they seek in their work and the characteristics of their current professional role or training context. Responses were rated on a seven-point Likert-type scale ranging from 1 (*not at all*) to 7 (*very much*).

#### 2.2.3 Work satisfaction

Work satisfaction was assessed using a single-item measure (42). Participants were asked to rate their overall satisfaction with their work. Responses were rated on a seven-point Likert-type scale ranging from 1 (*not satisfied at all*) to 7 (*very satisfied*).

#### 2.2.4 Utrecht work engagement scale-9 (UWES-9)

The UWES-9 (43) is a nine-item questionnaire that assesses the level of individuals' work engagement (e.g., “At my work, I feel bursting with energy”). The UWES-9 comprises three subscales: vigor, dedication, and absorption. Each subscale consists of three items, presented on a seven-point Likert-type scale, ranging from 0 (*never*) to 6 (*always*). Testing samples from 10 countries found that the 1-year test-retest reliabilities for the total UWES-9 score exceeded *r* = 0.56, and the internal consistency reliabilities (Cα) were above 0.85 in all countries (43, 44). In the present investigation, Cronbach's α was 0.91 for physicians, 0.90 for residents, 0.89 for students, and 0.90 overall.

#### 2.2.5 The burnout assessment tool (BAT)

The short version of the BAT (16, 45) consists of 12 statements assessing the degree of work burnout (e.g., “At work, I feel mentally exhausted”). It addresses four dimensions: exhaustion, mental distance, cognitive impairment, and emotional impairment, with each dimension represented by three items. Participants were asked to indicate how often each item applied to them on a scale from 0 (*never*) to 4 (*always*). Schaufeli et al. (16) reported a high internal consistency reliability of 0.95 for the overall scale. In the present investigation, Cronbach's α was 0.85 for physicians, 0.88 for residents, 0.88 for students, and 0.87 overall.

#### 2.2.6 Turnover intentions

Turnover intentions were assessed using a single-item measure (46). Participants were asked to indicate how often they had seriously considered leaving the medical profession. Responses were rated on a six-point Likert-type scale ranging from 0 (*never*) to 5 (*almost every day*).

#### 2.2.7 Commitment to the profession

Commitment to the profession was assessed using a single-item measure (47). Participants were asked whether they would choose a career in medicine again if given the opportunity to decide anew. Responses were rated on a six-point Likert-type scale ranging from 0 (*definitely not*) to 5 (*definitely yes*).

#### 2.2.8 Demographic questionnaire

Participants were asked to report their age, religion (Jewish, Muslim, Christian, Druze, or other), how religious they are ranging from 1 (*not at all*) to 7 (*very much*), their marital status (single, married, divorced, widowed, or other), whether they have children and if so how many, their native language (Hebrew, Arabic, Russian, English, French, or other), their socioeconomic status ranging from 1 (*very low*) to 7 (*very high*), and to rate how stressful their work as a physician is from 1 (*not at all*) to 7 (*very much*). In addition, group-specific information was collected. Physicians and residents reported their years of professional experience, medical specialty, and employment status (full-time or part-time). Medical students reported the type of program in which they were enrolled (4- or a 6-year program) and their current academic year.

### 2.3 Procedure

The present investigation employed a cross-sectional convenience sample. This investigation was approved by the Tel Aviv University ethics committee (#0006614-2). Data were collected via an online questionnaire. First, participants were provided with a brief description of the survey and gave their informed consent. They were then presented with a screening item to determine eligibility: “This survey is intended for physicians, residents, and medical students in their clinical years. What is your status?” Response options included: physician, resident, medical student in their clinical years, medical student not in their clinical years, or other. Individuals who selected “medical student not in their clinical years” or “other” were automatically redirected out of the survey and excluded from participation.

Eligible participants first reported their gender, which was used to tailor Hebrew pronouns throughout the survey. They then completed the following measures in a fixed order: the work orientation questionnaire, the person-environment fit item, the work satisfaction item, the work engagement scale, the burnout scale, the turnover intentions item, followed by the commitment to the profession item. Medical students were instructed to respond to the items based on their general perception of the medical profession and/or their clinical training experience to date. Finally, all participants completed the demographic questionnaire.

### 2.4 Data analysis

Data analysis was performed using SPSS 21.0 (63). Group differences among physicians, residents, and clinical-year medical students in work orientations and work-related wellbeing indicators were examined using analyses of variance (ANOVAs). To test the hypotheses concerning the associations between work orientations and work-related wellbeing indicators, Pearson correlation analyses were conducted. All statistical tests were conducted with two-tailed α = 0.05.

## 3 Results

### 3.1 Work orientations by group

Table 1 presents the means and standard deviations of the research variables. We conducted a repeated-measures ANOVA with a Greenhouse-Geisser correction, with group (physicians, residents, and clinical-year medical students) as the between-subject factor and work orientations as the within-subject dependent variables. A main effect for work orientations was found, *F*_(3.60, 701.90)_ = 108.63, *p* < 0.001, $\eta_{p}^{2}$ = 0.36. Specifically, calling was the most strongly endorsed work orientation, followed by career, then social embeddedness and busyness, which did not differ from one another (*p* = 0.747), and then followed by job.

**Table 1:** Means and standard deviations of research variables (*N* = 198).

Group	Physicians (*n_1_* = 54)		Residents (*n_2_* = 62)		Medical students (*n_3_* = 82)	
**Variable**	***M***	***SD***	***M***	***SD***	***M***	***SD***
Work orientations	
Calling	5.40	1.22	5.33	1.02	5.75	0.90
Job	3.42	1.40	3.13	1.27	3.03	1.17
Career	4.59	1.50	4.95	1.17	5.17	1.18
Social embeddedness	3.92	1.08	4.14	1.30	4.50	1.02
Busyness	4.39	1.41	3.63	1.25	4.44	1.31
Work-related wellbeing	
Person-environment fit	5.39	1.38	5.06	1.47	5.18	0.96
Work satisfaction	5.24	1.37	4.79	1.58	4.90	0.92
Work engagement	4.37	1.05	4.33	1.05	4.44	0.83
Burnout	1.29	0.50	1.36	0.55	1.36	0.53
Turnover intentions	1.35	1.43	1.84	1.45	1.17	0.93
Commitment to the profession	3.44	1.53	3.06	1.59	3.88	1.06

There was also a main effect for group, *F*_(2, 195)_ = 5.58, *p* = 0.004, $\eta_{p}^{2}$ = 0.05. Medical students showed a higher overall work-orientation profile than physicians and residents. However, physicians and residents did not differ (*p* = 0.360).

We found a work orientation x group interaction, *F*_(7.20, 701.90)_ = 3.73, *p* < 0.001, $\eta_{p}^{2}$ = 0.04. In follow-up tests of simple effects, the calling orientation was higher for medical students compared to residents; however, physicians did not differ significantly from either residents or medical students, *F*_(2, 195)_ = 3.46, *p* = 0.033, $\eta_{p}^{2}$ = 0.03. The career orientation was higher for medical students compared to physicians; however, residents did not differ from both physicians and medical students, *F*_(2, 195)_ = 3.37, *p* = 0.036, $\eta_{p}^{2}$ = 0.03. The social embeddedness orientation was higher for medical students compared to physicians; however, residents did not differ from both physicians and medical students, *F*_(2, 195)_ = 4.66, *p* = 0.011, $\eta_{p}^{2}$ = 0.05. The busyness orientation was lower for residents compared to physicians and medical students; however, physicians and medical students did not differ, *F*_(2, 195)_ = 7.64, *p* = 0.001, $\eta_{p}^{2}$ = 0.07. In job orientation, there was no significant difference among the groups (*p* = 0.207).

### 3.2 Work-related wellbeing indicators by group

We conducted a series of one-way ANOVAs with group (physicians, residents, and clinical-year medical students) as the between-subject factor and each work-related wellbeing indicator as the dependent variable. There were no group differences in person-environment fit (*p* = 0.377), work satisfaction (*p* = 0.150), work engagement (*p* = 0.782), or burnout (*p* = 0.698). However, there was a significant difference among groups in turnover intentions, *F*_(2, 195)_ = 5.16, *p* = 0.007, $\eta_{p}^{2}$ = 0.05. *Post-hoc* comparisons revealed that residents reported higher turnover intentions than both physicians and medical students, who did not differ from each other. A significant group difference was also found in commitment to the profession, *F*_(2, 195)_ = 6.27, *p* = 0.002, $\eta_{p}^{2}$ = 0.06. Specifically, medical students reported higher commitment to the profession than residents, whereas physicians did not differ significantly from either group.

### 3.3 Associations between work orientations and indicators of work-related wellbeing

The pattern of the interrelations among the five work orientations and six indicators of work-related wellbeing was highly similar among the three groups: between physicians and residents, *r*_s_ = 0.87 (*p* < 0.001); between physicians and medical students, *r*_s_ = 0.87 (*p* < 0.001); and between residents and students, *r*_s_ = 0.83 (*p* < 0.001). Nevertheless, there were differences among the groups. Table 2 presents the Pearson correlations of work orientations and work-related wellbeing indicators, separately for the three groups. Table 3 summarizes the significant associations between work orientations and work-related wellbeing indicators and indicates whether the findings were consistent with the research hypotheses.

**Table 2:** Pearson correlations between work orientations and work-related wellbeing, by group (*N* = 198).

Group	Work orientation	Person-environment fit	Work satisfaction	Work engagement	Burnout	Turnover intentions	Commitment to the profession
Physicians (*n_1_* = 54)	Calling	**0.44** ^**^	**0.54** ^**^	**0.49** ^**^	−0.10	–0.47^**^	**0.52** ^**^
	Job	−0.05	−0.16	−0.12	−0.19	0.20	−0.21
	Career	0.25	0.18	0.25	0.09	−0.02	−0.05
	Social embeddedness	−0.10	0.01	0.25	−0.06	−0.04	0.03
	Busyness	0.21	0.10	**0.30** ^*^	−0.10	−0.12	0.16
Residents (*n_2_* = 62)	Calling	**0.30** ^*^	**0.29** ^*^	**0.33** ^**^	–0.30^*^	–0.27^*^	**0.33** ^**^
	Job	−0.09	−0.04	−0.06	0.06	−0.11	−0.21
	Career	−0.08	−0.09	0.06	−0.05	–0.26^*^	0.06
	Social embeddedness	**0.32** ^*^	**0.33** ^**^	**0.43** ^**^	−0.25	–0.26^*^	0.22
	Busyness	0.18	**0.29** ^*^	**0.28** ^*^	−0.07	−0.10	**0.29** ^*^
Medical students (*n_3_* = 82)	Calling	0.18	0.14	**0.45** ^**^	0.03	−0.13	**0.31** ^**^
	Job	−0.02	−0.13	–0.28^*^	0.06	**0.28** ^*^	–0.27^*^
	Career	0.08	0.15	0.07	−0.00	0.04	0.01
	Social embeddedness	−0.10	0.03	**0.27** ^*^	0.16	−0.07	0.08
	Busyness	0.05	0.14	**0.49** ^**^	−0.12	–0.26^*^	**0.28** ^*^

^*^p < 0.05, ^**^p < 0.01. Significant associations are in **bold**.

**Table 3:** Summary of hypothesized and observed associations between work orientations and work-related wellbeing by group.

Group	Calling	Job	Career	Social embeddedness	Busyness
Physicians	**+** **Person-environment fit** **+** **Work satisfaction** **+** **Work engagement** – **Turnover intentions** + Commitment to the profession				+ Work engagement
Residents	**+** **Person-environment fit** **+** **Work satisfaction** **+** **Work engagement** – **Burnout** – **Turnover intentions** + Commitment to the profession		– Turnover intentions	+ Person-environment fit **+** **Work satisfaction** **+** **Work engagement** – Turnover intentions	+ Work satisfaction + Work engagement + Commitment to the profession
Medical students	**+** **Work engagement** + Commitment to the profession	– **Work engagement** + Turnover intentions – Commitment to the profession		**+** **Work engagement**	+ Work engagement – Turnover intentions + Commitment to the profession

(+) indicates a positive association; (–) indicates a negative association. Significant hypothesized associations are shown in **bold**.

#### 3.3.1 Physicians

Among physicians, only two work orientations were significantly associated with work-related wellbeing indicators: calling and busyness. Specifically, a higher calling orientation was associated with greater person-environment fit, work satisfaction, work engagement, commitment to the profession, and lower turnover intentions. Additionally, a higher busyness orientation was associated with greater work engagement.

#### 3.3.2 Residents

Among residents, four work orientations (excluding job orientation) were significantly associated with work-related wellbeing indicators. A higher calling orientation was associated with greater person-environment fit, work satisfaction, work engagement, commitment to the profession, and lower burnout and turnover intentions. A higher social embeddedness orientation was associated with greater person-environment fit, work satisfaction, work engagement, and lower turnover intentions. A higher busyness orientation was associated with greater work satisfaction, work engagement, and commitment to the profession. Finally, a higher career orientation was associated with lower turnover intentions.

#### 3.3.3 Medical students

Among medical students, four work orientations (excluding career orientation) were significantly associated with work-related wellbeing indicators. A higher calling orientation was associated with greater work engagement and stronger commitment to the profession. A higher job orientation was associated with lower work engagement and commitment to the profession, as well as higher turnover intentions. A higher busyness orientation was associated with greater work engagement and commitment to the profession, as well as lower turnover intentions. Finally, a higher social embeddedness orientation was associated with greater work engagement.

## 4 Discussion

The present investigation examined and compared physicians, residents, and clinical-year medical students regarding their work orientations, work-related wellbeing, and the associations between these constructs. Comparing these groups at different professional stages, alongside a broad set of work orientations and work-related wellbeing indicators, provides a comprehensive understanding of how work orientations relate to work-related wellbeing in this highly demanding field.

### 4.1 Differences in work orientations and work-related wellbeing in medicine

The present investigation's findings indicated a distinct hierarchy of work orientations in medicine, with calling orientation emerging as the most strongly endorsed, followed by career, social embeddedness and busyness, and job orientations. Previous research in the general population, conducted by Willner et al. (2), found career orientation to be the most endorsed orientation. This pattern may suggest that calling may play a particularly prominent role in the medical profession. These results align with previous empirical evidence highlighting the strong presence of calling in the medical field. For example, Duffy and Sedlacek (48) found that students aspiring to obtain a medical degree were more likely than those with other occupational aspirations to endorse a calling orientation. In a survey of U.S. primary care physicians and psychiatrists, approximately 40% reported a strong sense of calling (49). Similarly, Borges et al. (50) found that even first-year medical students reported this, with 55% indicating a strong sense of medicine as a calling. Manuel et al. (51) further reported that medical students were significantly more likely to report a calling orientation than their counterparts in dentistry or pharmacy.

Interestingly, we found that clinical-year medical students showed a different pattern of work orientations than physicians and residents, consistent with the exploratory nature of individuals at this stage of professional development in medicine. Specifically, medical students reported higher calling than residents, and higher career and social embeddedness than physicians. However, residents exhibited the lowest busyness orientation, possibly reflecting workload strain during residency (52). Nevertheless, job orientation was stable across groups. Medical students in their clinical years may hold stronger calling, career, and social embeddedness orientations because they are still developing their professional identity and view medicine through an aspirational lens. In contrast, residents and physicians, who are more exposed to the demands and constraints of medical practice, may recalibrate these orientations over time. These findings are consistent with previous research, which has shown that factors such as calling (53) and occupational aspirations (54) tend to decline during the transition from study to work across various professional fields.

Finally, no group difference emerged across several indicators of work-related wellbeing (i.e., person-environment fit, work satisfaction, work engagement, and burnout). This finding suggests broadly similar levels of these indicators across groups, although interpretation should be cautious given differences in training stage and role experience. Nevertheless, notable differences emerged for turnover intentions and commitment to the profession. Residents reported significantly higher turnover intentions than physicians and medical students. This finding is consistent with evidence that residency is often a period of heightened stress, workload strain, and career uncertainty (55, 56), which may prompt reconsideration of long-term career plans. A group difference also emerged in commitment to the profession, with medical students reporting higher commitment than residents, whereas physicians did not differ significantly from either group. This pattern suggests that commitment to the profession may fluctuate across stages of medical training rather than increase linearly, with residency perhaps representing a particularly challenging period in this respect. Together, these findings suggest residency may represent a particularly vulnerable stage for retention, underscoring the importance of targeted support to maintain commitment and reduce attrition risk during this transition period.

### 4.2 Associations of work orientations with work-related wellbeing

Overall, we found that calling was the orientation most consistently associated with better work-related wellbeing across the medical profession, whereas the relevance of other orientations, particularly social embeddedness and busyness, varied by stage of professional development. In the following sections, we discuss each orientation in turn.

#### 4.2.1 Calling orientation

A stronger calling orientation was associated with greater work engagement across all three groups. This finding supports our hypothesis and aligns with previous evidence demonstrating an association between calling and work engagement in the general population (4, 5). Moreover, we found that a stronger calling orientation was associated with commitment to the profession in all three groups. Among physicians and residents, but not students, a stronger calling orientation was associated with better person-environment fit, greater work satisfaction, and lower turnover intentions. These findings are consistent with our hypotheses and with previous studies conducted in the general population (2, 31), among other healthcare professionals such as nurses (32, 35) and among physicians (36). These associations did not emerge among medical students, possibly because their clinical experience was limited, which may have reduced their ability to make meaningful evaluations of fit, satisfaction, or turnover intentions. Finally, a calling orientation was associated with lower burnout only among residents, supporting our hypothesis and consistent with previous research among healthcare professionals (34) and in the general population (33, 34). This suggests that maintaining a sense of calling during residency may help protect against emotional exhaustion during this particularly demanding stage of medical training.

#### 4.2.2 Job orientation

Among medical students, a stronger job orientation was associated with lower work engagement, supporting our hypothesis and consistent with previous findings in the general population (4, 5). We also found that, among medical students, a stronger job orientation was associated with greater turnover intentions and lower commitment to the profession. This contradicts previous research reporting no association between job orientation and turnover intentions in the general population (25). One possible explanation is that job-oriented medical students enter training with heightened expectations of income, discover that they will not necessarily be met, thereby increasing their intentions of leaving the profession. Job orientation was not associated with work satisfaction for any of the three groups, contrary to our hypothesis and evidence from the general population (2, 4, 5). Moreover, among physicians and residents, job orientation showed no association with any work-related wellbeing indicator, suggesting that in later career stages, external motivators such as salary or job security may be less relevant to wellbeing than earlier stages of the medical training.

#### 4.2.3 Career orientation

Career orientation was generally not associated with work-related wellbeing across the three groups. Some of these null associations are consistent with findings in the general population (2, 4). One possible explanation is that career advancement is inherently embedded in the medical profession and, as such, does not function as either a protective or risk factor for work-related wellbeing. Only one significant association emerged regarding career orientation. Among residents, a higher career orientation was associated with lower turnover intentions. This negative association was unexpected, as prior research in the general population has demonstrated a positive association (25). A possible explanation is that residents perceive the medical profession as offering opportunities for career advancement, which may not reflect the experience in the general population, thereby decreasing their intentions to leave the profession.

#### 4.2.4 Social embeddedness orientation

Among residents and medical students (but not physicians), a stronger social embeddedness orientation was associated with greater work engagement, supporting our hypothesis and in line with previous findings in the general population (4, 5). This pattern may reflect medical students' and residents' greater reliance on workplace relationships as sources of support and belonging. In turn, this reliance may enhance their engagement, whereas physicians may draw on these resources outside of work. Among residents alone (but not students or physicians), a stronger social embeddedness orientation was associated with better person-environment fit, greater work satisfaction, and lower turnover intentions. The association with work satisfaction supports our hypothesis and aligns with previous findings in the general population (2, 4). Here again, the demanding workload and the long hours of residency may limit opportunities to cultivate relationships outside of work, making social embeddedness orientation within the professional environment particularly salient for predicting work-related wellbeing. Contrary to our hypothesis and previous findings among nurses (39), social embeddedness orientation was not associated with burnout in all three groups. One explanation may be that the intense and persistent demands of medical work overshadow the potential protective effects of social embeddedness, thereby diminishing its association with burnout.

#### 4.2.5 Busyness orientation

A greater busyness orientation was associated with higher work engagement across all groups. Although this was not part of our hypothesis, it is consistent with some previous findings in the general population (5). Among residents and medical students (but not physicians), a stronger busyness orientation was also associated with a higher commitment to the profession. Among residents alone, a higher busyness orientation was associated with greater work satisfaction, contradicting findings in the general population that showed a negative association (2). This may indicate that, for residents, being busy is a source of satisfaction, perhaps reflecting the learning-intensive nature of this stage of professional development. In addition, among medical students alone, this orientation was associated with fewer turnover intentions. These associations suggest that a hectic lifestyle is more highly valued in the early stages of the medical profession than in its later stages, potentially serving as a protective factor for wellbeing.

### 4.3 Practical implications

The present investigation's findings indicated that work orientations can serve as either a resilience or risk factor in promoting or hindering work-related wellbeing in medicine. This highlights their potential as targets for interventions, as workers' perceptions of their work matter (57). First, calling orientation showed the most consistent positive pattern of associations with work-related wellbeing indicators across stages of the medical profession. Accordingly, interventions that nurture and sustain a sense of calling may enhance engagement, satisfaction, and commitment to the profession while reducing burnout and turnover intentions. Evidence from West et al. (58) shows that a facilitated small-group intervention, integrating collegiality, shared reflection, and skills in self-awareness and mindfulness, enhanced physicians' sense of meaning and engagement. At the same time, it reduced depersonalization, offering a practical model for reinforcing calling orientation and sustaining wellbeing.

Second, the associations between work orientations and work-related wellbeing varied by professional development stage. For example, social embeddedness and busyness orientations were more strongly related to favorable work-related wellbeing indicators among residents and medical students than among physicians. This suggests that interventions should be stage-specific, reinforcing relevant orientations during vulnerable transitions, such as residency, when work-related wellbeing may decline due to unique challenges that may arise in this stage (59).

Furthermore, educational and organizational policies should recognize the shifting relevance of work orientations over time and provide tailored support accordingly. For instance, as demonstrated in previous studies, fostering meaningful interpersonal connections and a sense of belonging could strengthen engagement, particularly during the early stages of professional development, such as medical school and residency (60, 61). Therefore, policies should include clear orientation processes, adjusted schedules, efficient workflow maintenance, and dedicated teaching time to support both engagement and efficiency in high-paced environments. To ensure this remains adaptive rather than overwhelming, structured integration of such policies into busy clinical practices is essential.

### 4.4 Limitations and future research

The present investigation has several limitations. First, its cross-sectional design precludes causal inferences about whether work orientations affect work-related wellbeing or vice versa. Moreover, we found differences in work orientations and their associations with work-related wellbeing across stages of the medical profession. Future research can employ experimental or longitudinal designs to test the directionality of these associations and to trace their development over time. Such findings would inform future interventions tailored to causal mechanisms and developmental stages.

Second, given the limited availability of physicians and prospective physicians, we employed a convenience sample, resulting in relatively small group sizes and potential selection bias. Nevertheless, the samples demonstrated substantial variability in both work orientations and work-related wellbeing, both between and within groups, with each group comprising more than 50 participants. Future research should aim to replicate these findings in larger, more representative samples using mandatory or systematic data collection procedures. This would enhance the generalizability of the practical implications derived from this research. Third, the present investigation relied only on self-report measures. Although such measures are appropriate and valid for assessing subjective constructs such as work-related wellbeing, they may introduce shared method variance and rely on participants' subjective perceptions. Future research should incorporate behavioral or observational indicators.

Finally, although the internal consistency estimates for most WOQ short-form dimensions were acceptable, the reliability coefficients for two dimensions (0.67 for calling and 0.68 for social embeddedness) fell slightly below the conventional 0.70 threshold. At the subgroup level, several additional coefficients were also below this threshold, including calling among residents (0.51) and students (0.68), career among residents (0.68), and social embeddedness among physicians (0.57). This might be an artifact of the small group sample sizes (62). Alternatively, they may indicate that certain WOQ items function somewhat differently across respondents at different stages of medical training, possibly as a function of professional socialization. Future research should examine this possibility directly. Accordingly, findings involving these orientations should be interpreted with caution. Future research is also needed to examine the psychometric properties of the short-form WOQ in medical populations.

### 4.5 Conclusion

This investigation examined the associations between work orientations and work-related wellbeing across different stages of the medical profession. The findings contribute to the growing literature on the extended model of work orientations and its associations with work-related wellbeing within the medical field. Specifically, a calling orientation was generally associated with better work-related wellbeing. Among residents and medical students, social embeddedness and busyness orientations were also associated with several favorable indicators of work-related wellbeing. In contrast, among medical students, a job orientation was associated with poorer work-related wellbeing. Overall, these findings suggest that work orientations and their associations with work-related wellbeing evolve across different stages of the medical profession.

## Data Availability

The raw data supporting the conclusions of this article will be made available by the authors, without undue reservation.

## References

[1] Hirschi A. The fourth industrial revolution: issues and implications for career research and practice. Career Dev Q. (2018) 66:192–204. doi: 10.1002/cdq.12142

[2] Willner T Lipshits-Braziler Y Gati I. Construction and initial validation of the work orientation questionnaire. J Career Assess (2020) 28:109–27. doi: 10.1177/1069072719830293

[3] Willner T Lipshits-Braziler Y Gati I Shachrur B. Testing the validity of the expanded five-dimensional model of work orientations. J Career Assess (2024) 32:721–44. doi: 10.1177/10690727241232437

[4] Lipshits-Braziler Y Abessolo M Santilli S Di Maggio I. Work orientation questionnaire: measurement invariance and criterion validity among Swiss, Israeli, and Italian workers. Int J Educ Vocat Guid. (2021) 21:187–209. doi: 10.1007/s10775-020-09436-1

[5] Liu D Wang Y Li C. Development and validation of the work orientation questionnaire short-form (WOQ-SF): evidence from China. Eur J Psychol Assess (2025) 41:402–13. doi: 10.1027/1015-5759/a000814

[6] Houseknecht VE Roman B Stolfi A Borges NJ. A longitudinal assessment of professional identity, wellness, imposter phenomenon, and calling to medicine among medical students. Med Sci Educ. (2019) 29:493–7. doi: 10.1007/s40670-019-00718-0 34457506 PMC8368953

[7] Jager AJ Tutty MA Kao AC. Association between physician burnout and identification with medicine as a calling. Mayo Clin Proc. (2017) 92:415–22. doi: 10.1016/j.mayocp.2016.11.012 28189341

[8] Bellah RN Higham J Madsen R Sullivan WM Swidler A Tipton SM. Habits of the Heart: Individualism and Commitment in American Life. Berkeley, CA: University of California Press (1985).

[9] Wrzesniewski A McCauley C Rozin P Schwartz B. Jobs, careers, and callings: people's relations to their work. J Res Pers. (1997) 31:21–33. doi: 10.1006/jrpe.1997.2162

[10] Kristof-Brown AL Zimmerman RD Johnson EC. Consequences of individuals' fit at work: a meta-analysis of person-job, person-organization, person-group, and person-supervisor fit. Pers Psychol. (2005) 58:281–342. doi: 10.1111/j.1744-6570.2005.00672.x

[11] Locke EA. What is job satisfaction? Organ Behav Hum Perform. (1969) 4:309–36. doi: 10.1016/0030-5073(69)90013-0

[12] Wright TA Cropanzano R. Psychological well-being and job satisfaction as predictors of job performance. J Occup Health Psychol. (2000) 5:84–94. doi: 10.1037//1076-8998.5.1.84 10658888

[13] Zhang S Wang J Xie F Yin D Shi Y Zhang M . A cross-sectional study of job burnout, psychological attachment, and the career calling of Chinese doctors. BMC Health Serv Res. (2020) 20:1–9. doi: 10.1186/s12913-020-4996-y PMC7068889 32164684

[14] Schaufeli WB Salanova M Bakker AB Gonzales-Roma V. The measurement of engagement and burnout: a two sample confirmatory factor analytic approach. J Happiness Stud. (2002) 3:71–92. doi: 10.1023/A:1015630930326

[15] Bakker AB Demerouti E. Towards a model of work engagement. Career Dev Int. (2008) 13:209–23. doi: 10.1108/13620430810870476

[16] Schaufeli WB Desart S De Witte H. Burnout assessment tool (BAT)-development, validity, and reliability. Int J Environ Res Public Health (2020) 17:9495. doi: 10.3390/ijerph17249495 33352940 PMC7766078

[17] Schaufeli W de Witte H. Burnout assessment tool (BAT). In:Krägeloh CU Alyami M Medvedev ON, editors. International Handbook of Behavioral Health Assessment. Cham: Springer (2023). p. 1–24. doi: 10.1007/978-3-030-89738-3_54-1

[18] Hann M Reeves D Sibbald B. Relationships between job satisfaction, intentions to leave family practice and actually leaving among family physicians in England. Eur J Public Health (2011) 21:499–503. doi: 10.1093/eurpub/ckq005 20142402

[19] Coladarci T. Teachers' sense of efficacy and commitment to teaching. J Exp Educ. (1992) 60:323–37. doi: 10.1080/00220973.1992.9943869

[20] Chambel MJ Carvalho VS. Commitment and wellbeing: the relationship dilemma in a two-wave study. Front Psychol. (2022) 13:816240. doi: 10.3389/fpsyg.2022.816240 35465506 PMC9029816

[21] Firth-Cozens J. Interventions to improve physicians' well-being and patient care. Soc Sci Med. (2001) 52:215–22. doi: 10.1016/S0277-9536(00)00221-5 11144777

[22] Shanafelt TD West CP Sinsky C Trockel M Tutty M Satele DV . Changes in burnout and satisfaction with work-life integration in physicians and the general US working population between 2011 and 2017. Mayo Clin Proc. (2019) 94:1681–94. doi: 10.1016/j.mayocp.2018.10.023 30803733

[23] Shanafelt TD Dyrbye LN West CP Trockel M Tutty M Wang H . Career plans of US physicians after the first 2 years of the COVID-19 pandemic. Mayo Clin Proc. (2023) 98:1629–40. doi: 10.1016/j.mayocp.2023.07.006 37923521

[24] Tyssen R Hem E Gude T Grønvold NT Ekeberg Ø Vaglum P. Lower life satisfaction in physicians compared with a general population sample: a 10-year longitudinal, nationwide study of course and predictors. Soc Psychiatry Psychiatr Epidemiol. (2009) 44:47–54. doi: 10.1007/s00127-008-0403-4 18642123

[25] Nikolova M. Loud or Quiet Quitting? The Influence of Work Orientations on Effort and Turnover. (2024). Available online at: http://www.iza.org (Accessed April 6, 2025).

[26] Ali Jadoo SA Aljunid SM Dastan I Tawfeeq RS Mustafa MA Ganasegeran K . Job satisfaction and turnover intention among Iraqi doctors–a descriptive cross-sectional multicentre study. Hum Resour Health (2015) 13:21. doi: 10.1186/s12960-015-0014-6 25903757 PMC4407309

[27] Kumar S. Burnout and doctors: prevalence, prevention and intervention. Healthcare (2016) 4:37. doi: 10.3390/healthcare4030037 27417625 PMC5041038

[28] Van Ham I Verhoeven AH Groenier KH Groothoff JW De Haan J. Job satisfaction among general practitioners: a systematic literature review. Eur J Gen Pract. (2006) 12:174–80. doi: 10.1080/13814780600994376 17127604

[29] Cohen Aubart F Lhote R Steichen O Roeser A Otriv N Levesque H . Workload, well-being and career satisfaction among French internal medicine physicians and residents in 2018. Postgrad Med J. (2020) 96:21–7. doi: 10.1136/postgradmedj-2019-136657 31467142

[30] Dyrbye LN West CP Satele D Boone S Tan L Sloan J. et al. Burnout among US medical students, residents, and early career physicians relative to the general US population. Acad Med. (2014) 89:443–51. doi: 10.1097/ACM.0000000000000134 24448053

[31] Xu Y Liu K Chen K Feng M. How does person-environment fit relate to career calling? The role of psychological contracts and organizational career management. Psychol Res Behav Manag. (2023) 16:1597–609. doi: 10.2147/PRBM.S404374 37159647 PMC10163889

[32] Xu S Tao L Huang H Little J Huang L. Pediatric nurses' turnover intention and its association with calling in China's tertiary hospitals. J Pediatr Nurs. (2020) 52:e51–6. doi: 10.1016/j.pedn.2020.01.005 32007328

[33] Da Silva Filho AFM Felix B Mainardes EW. Occupational callings: a double-edged sword for burnout and stress. Estud Psicol. (2021) 26:45–55. doi: 10.22491/1678-4669.20210006

[34] Goštautaite B Bučiuniene I Dalla Rosa A Duffy R Kim HJ. Healthcare professionals with calling are less likely to be burned out: the role of social worth and career stage. Career Dev Int. (2020) 25:649–70. doi: 10.1108/CDI-10-2018-0255

[35] Hong Y Qiu M Liu L Huang F Wang K Lin R. Surface acting, emotional exhaustion, career calling and turnover intention among student nurses: a cross-sectional study. Nurse Educ Pract. (2023) 69:103641. doi: 10.1016/j.nepr.2023.103641 37060732

[36] Bott EM Duffy RD Borges NJ Braun TL Jordan KP Marino JF. Called to medicine: physicians' experiences of career calling. Career Dev Q. (2017) 65:113–30. doi: 10.1002/cdq.12086

[37] Lazar A Littman-Ovadia H Ovadia T. Medicine as a calling among male and female medical students in Israel: does it make a difference? Int J Educ Vocat Guid. (2018) 18:279–95. doi: 10.1007/s10775-018-9361-x

[38] Creed PA Rogers ME Praskova A Searle J. Career calling as a personal resource moderator between environmental demands and burnout in Australian junior doctors. J Career Dev. (2014) 41:547–61. doi: 10.1177/0894845313520493

[39] Van Yperen NW Buunk BP Schaufeli WB. Communal orientation and the burnout syndrome among nurses. J Appl Soc Psychol. (1992) 22:173–89. doi: 10.1111/j.1559-1816.1992.tb01534.x

[40] Faul F Erdfelder E Lang AG Buchner A. G^*^Power 3: a flexible statistical power analysis program for the social, behavioral, and biomedical sciences. Behav Res Methods (2007) 39:175–91. doi: 10.3758/BF03193146 17695343

[41] Willner T Lipshits-Braziler Y Gati I. Development and validation of an 18-item short form of the Work Orientation Questionnaire. Unpublished manuscript. Jerusalem: The Hebrew University of Jerusalem (2023).

[42] Dolbier CL Webster JA McCalister KT Mallon MW Steinhardt MA. Reliability and validity of a single-item measure of job satisfaction. Am J Health Promot. (2005) 19:194–8. doi: 10.4278/0890-1171-19.3.194 15693347

[43] Schaufeli WB Bakker AB Salanova M. The measurement of work engagement with a short questionnaire: a cross-national study. Educ Psychol Meas. (2006) 66:701–16. doi: 10.1177/0013164405282471

[44] Littman-Ovadia H Balducci C. Psychometric properties of the Hebrew version of the Utrecht work engagement scale (UWES-9). Eur J Psychol Assess (2013) 29:58–63. doi: 10.1027/1015-5759/a000121

[45] HadŽibajramović E Schaufeli W De Witte H. Shortening of the burnout assessment tool (BAT)—from 23 to 12 items using content and Rasch analysis. BMC Public Health (2022) 22:560. doi: 10.1186/s12889-022-12946-y 35313849 PMC8939057

[46] Spector PE. Measurement of human service staff satisfaction: development of the job satisfaction survey. Am J Community Psychol. (1985) 13:693–713. doi: 10.1007/BF00929796 4083275

[47] Fresko B Kfir D Nasser F. Predicting teacher commitment. Teach Teach Educ. (1997) 13:429–38. doi: 10.1016/S0742-051X(96)00037-6

[48] Duffy RD Sedlacek WE. The salience of a career calling among college students: exploring group differences and links to religiousness, life meaning, and life satisfaction. Career Dev Q. (2010) 59:27–41. doi: 10.1002/j.2161-0045.2010.tb00128.x

[49] Yoon JD Shin JH Nian AL Curlin FA. Religion, sense of calling, and the practice of medicine: findings from a national survey of primary care physicians and psychiatrists. South Med J. (2015) 108:189–95. doi: 10.14423/SMJ.0000000000000250 25772054 PMC4452021

[50] Borges NJ Manuel RS Duffy RD. Speciality interests and career calling to medicine among first-year medical students. Perspect Med Educ. (2013) 2:14–7. doi: 10.1007/S40037-012-0037-9 23670652 PMC3576487

[51] Manuel RS Borges NJ Adcock K Smith J. Professional identity and career calling across medical, pharmacy, and dental students. Med Sci Educ. (2018) 28:19–21. doi: 10.1007/s40670-017-0508-z

[52] Temple J. Resident duty hours around the globe: where are we now? BMC Med Educ. (2015) 15:S8. doi: 10.1186/1472-6920-14-S1-S8 25559277 PMC4304287

[53] Rosa AD Gerdel S Vianello M. What happened to your calling? The development of calling across college-to-work transition. J Career Assess (2024) 32:407–26. doi: 10.1177/10690727231200259

[54] Lipshits-Braziler Y Habayib H Cinamon RG. Career aspirations of Arab minority college students in Israel: a longitudinal study. J Career Dev. (2025) 52:301–19. doi: 10.1177/08948453251313796

[55] Stucky ER Dresselhaus TR Dollarhide A Shively M Maynard G Jain S . Intern to attending: assessing stress among physicians. Acad Med. (2009) 84:251–7. doi: 10.1097/ACM.0b013e3181938aad 19174680

[56] Zhou AY Panagioti M Esmail A Agius R Van Tongeren M Bower P. Factors associated with burnout and stress in trainee physicians: a systematic review and meta-analysis. JAMA Netw Open (2020) 3:e2013761. doi: 10.1001/jamanetworkopen.2020.13761 32809031 PMC7435345

[57] Ettner SL Grzywacz JG. Workers' perceptions of how jobs affect health: a social ecological perspective. J Occup Health Psychol. (2001) 6:101–13. doi: 10.1037//1076-8998.6.2.101 11326723

[58] West CP Dyrbye LN Rabatin JT Call TG Davidson JH Multari A . Intervention to promote physician well-being, job satisfaction, and professionalism: a randomized clinical trial. JAMA Intern Med. (2014) 174:527–33. doi: 10.1001/jamainternmed.2013.14387 24515493

[59] Hurst C Kahan D Ruetalo M Edwards S. A year in transition: a qualitative study examining the trajectory of first year residents' well-being. BMC Med Educ. (2013) 13:96. doi: 10.1186/1472-6920-13-96 23842470 PMC3712006

[60] Gold JA Bentzley JP Franciscus AM Forte C De Golia SG. An intervention in social connection: medical student reflection groups. Acad Psychiatry (2019) 43:375–80. doi: 10.1007/s40596-019-01058-2 30963416

[61] Ziegelstein RC. Creating structured opportunities for social engagement to promote well-being and avoid burnout in medical students and residents. Acad Med. (2018) 93:537–9. doi: 10.1097/ACM.0000000000002117 29280756

[62] Ikhsanudin I Subali B Retnawati H Istiyono E. Estimation of Cronbach reliability based on sample size, gender, and the grades. Int J Eval Res Educ. (2024) 13:759–66. doi: 10.11591/ijere.v13i2.24895

[63] IBM Corp. IBM SPSS Statistics for Windows, Version 21.0 (21). IBM Corp. (2012).

